# Carrageenans as a New Source of Drugs with Metal Binding Properties

**DOI:** 10.3390/md8041106

**Published:** 2010-04-01

**Authors:** Yuri S. Khotimchenko, Elena V. Khozhaenko, Maxim Y. Khotimchenko, Elena A. Kolenchenko, Valeri V. Kovalev

**Affiliations:** 1 Laboratory of Pharmacology, Institute of Marine Biology, Far East Branch of Russian Academy of Sciences, Vladivostok, 690041, Russia; E-Mail: eakolenchenko@mail.ru (E.A.K.); 2 Department of Pharmacology, Vladivostok State Medical University, Vladivostok, 690990, Russia; E-Mail: maxkhot@yandex.ru (M.Y.K.)

**Keywords:** carrageenan, heavy metal, metal binding activity, equilibrium study

## Abstract

Carrageenans are abundant and safe non-starch polysaccharides exerting their biological effects in living organisms. Apart from their known pro-inflammation properties and some pharmacological activity, carrageenans can also strongly bind and hold metal ions. This property can be used for creation of the new drugs for elimination of metals from the body or targeted delivery of these metal ions for healing purposes. Metal binding activity of different carrageenans in aqueous solutions containing Y^3+^ or Pb^2+^ ions was studied in a batch sorption system. The metal uptake by carrageenans is not affected by the change of the pH within the range from 2.0 to 6.0. The rates and binding capacities of carrageenans regarding metal ions were evaluated. The Langmuir, Freundlich and BET sorption models were applied to describe the isotherms and constants, and the sorption isothermal data could be explained well by the Langmuir equation. The results obtained through the study suggest that κ-, ι-, and λ-carrageenans are favorable sorbents. The largest amount of Y^3+^ and Pb^2+^ ions are bound by ι-carrageenan. Therefore, it can be concluded that this type of polysaccharide is the more appropriate substance for elaboration of the drugs with high selective metal binding properties.

## 1. Introduction

Carrageenans are sulfated galactans containing D-galactose and its derivatives linked to each other by dint of repeating β(1→4) and α(1→3) bonds [1]. They occur in the cell wall and intercellular matrices of certain marine red algae. Three carrageenans derivatives, κ-, ι-, and λ-, are widely used in the food industry as thickening, gelling, and protein-suspending agents [2]. On the other hand, carrageenans are commonly used in experimental studies as inflammation inducers as well as immune modifiers due to their selective toxic effects on macrophages [3]. Besides, several studies have shown the pharmacological activity of carrageenans. Some types of carrageenans were shown to exert anticoagulant effects [4], reduce blood cholesterol levels [5] and to possess antiviral and antibacterial properties [6].

In addition carrageenans possess metal-binding properties, whereby they can be successfully used in various fields of medicine and pharmacy. Natural compounds exerting high selective activity regarding metal ions may be used for treatment and prevention of the chronic metal poisoning in humans. Besides, such compounds may be the base for elaboration of drugs purposed for targeted radioactive therapy of malignant tumors. From this point of view, it is of importance to investigate the metal binding activity of carrageenans regarding the most common environmental pollutants and dangerous radioactive substances. In our experiments we used Pb^2+^ and Y^3+^ ions as the *in vitro* models for investigation of the metal-binding properties of carrageenans.

Yttrium is a product of strontium degradation and it should be taken into account in safety assessments of nuclear waste underground repositories. From this point of view the substances with high metal binding activity are of interest for removal of yttrium ions from waste waters of the nuclear industry plants or from the body of nuclear industry workers. Besides, yttrium 90 is used for radionuclide therapy of cancer diseases [7]. Releases of Pb^2+^ ions to the environment have been increasing continuously as a result of industrial activity and technological development posing a significant threat to the environment and public health because of its toxicity, incremental accumulation in the food chain and persistence in the ecosystem [8].

It should be noted that this work is a fundamental study investigating the binding capacity of three types of carrageenans regarding Y^3+^ and Pb^2+^ ions. In the present study κ-, ι-, and λ-carrageenans were evaluated regarding their capacity to bind and hold the aforementioned ions under *in vitro* conditions. The influence of experimental conditions such as pH, agitation period, agitation rate and initial concentrations on the parameters of the binding process were investigated. The Langmuir, Freundlich and Brunauer-Emmett-Teller (BET) equations were used to fit the equilibrium isotherm. This information will be useful for further system design applications in procedures requiring translocation of lead and yttrium ions.

## 2. Results and Discussion

### 2.1. Effect of pH

**Figure 1:**
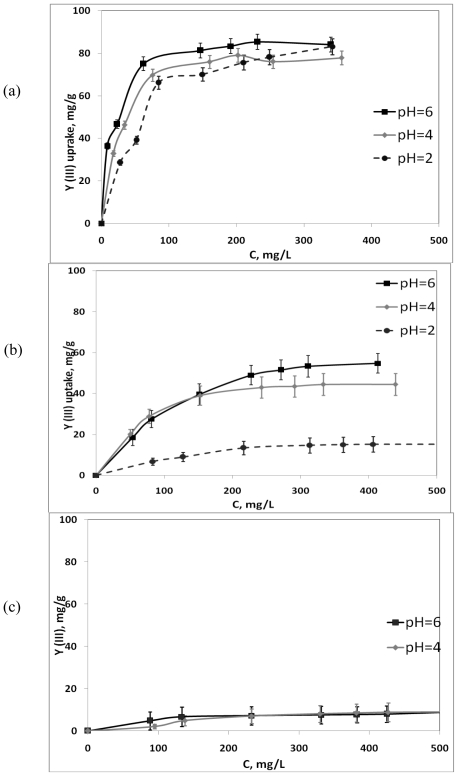
Yttrium binding activity of carrageenans under various pH values of solution (a) ι-carrageenan (b) κ-carrageenan (c) λ-carrageenan.

**Figure 2:**
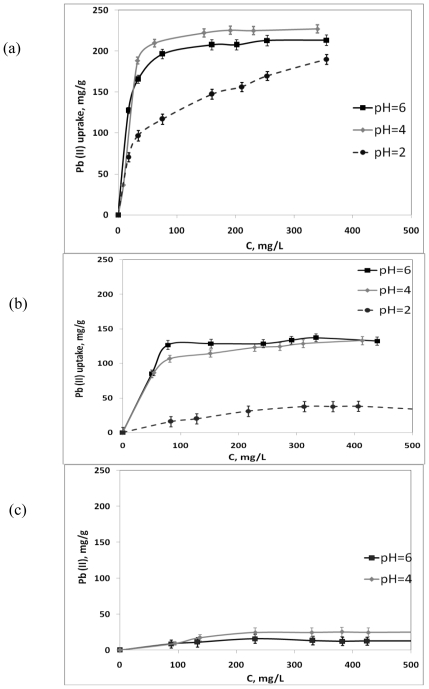
Lead binding activity of carrageenans under various pH values of solution (a) ι-carrageenan (b) κ-carrageenan (c) λ-carrageenan.

The rate of interaction between polysaccharides and metal ions is strongly pH dependent because properties (charge and potential) of both carrageenan compounds and the solution composition, *i.e.*, metal ion speciation, change according to the pH of the medium [9,10]. The number of active binding sites of the carrageenan molecule may also change accordingly with the varying pH. Therefore, estimation of the metal binding capacity of carrageenans in solutions with different pH values was performed at the beginning of the present study. Figures 1 and 2 depict the effect of the pH values on the Y^3+^ and Pb^2+^ uptake by the three types of carrageenans under studied.

It was established that the metal binding capacity of ι-carrageenan is significantly higher than that of other types of carrageenan. The activity of λ-carrageenan towards metal ions was almost negligible. It was found that binding activity of the ι-carrageenan sample regarding Pb^2+^ ions was significantly lower at pH 2.0 in comparison with the values obtained at pH 4.0 and 6.0. Presumably low pH would favor protonation of the active binding sulfate sites of the κ-carrageenan molecule resulting in a reversal of the charge of its molecule leading to the reduced metal binding activity of these types of polysaccharide. At the same time yttrium uptake of the ι-carrageenan sample was the same within the range of pH values from 2.0 to 6.0. It may be explained by the lower electrostatic forces of the bivalent lead ions in comparison with the trivalent yttrium ions. Due to stronger electrostatic interactions between the yttrium ions and active groups of carrageenan the binding process is not disrupted by protonation at the low pH values. It was observed that κ-carrageenan possesses lower binding activity, interacting with both metals studied at pH 2.0 and a significantly higher one at pH 4.0 and 6.0. Probably a lesser amount of sulfate groups contributes to the more important influence of protons on the spiral structure of κ-carrageenan. The structure of λ-carrageenan does not possess 3,6-anhydro rings and for this reason when this type of carrageenan interacts with metal ions it cannot form a spiral structure providing metal binding capacity, which is characteristic of ι-carrageenan and, to a smaller degree, of κ-carrageenan. Thus, the metal uptake capacity of λ-carrageenan was extremely low and this made it hard to determine any significant differences of its metal uptake capacity at the different pH values. In the media with pH values lower than 2.0 carrageenans as well as all non-starch polysaccharide substances are usually precipitated, and this sedimentation results in dramatic decrease of their binding activity [11]. At pH values higher than 6.0, metal binding capacity of all types of carrageenans studied was almost negligible due to the alkaline shift, which obviously contributes to the polysaccharides becoming unstable [10]. Moreover, ionic interactions between the components of the sorption system at such pH values must be disrupted due to the formation of hydro-complexes of Y^3+^ and Pb^2+^ ions. According to the literature [12], the chemical species of Y^3+^ existing in solution at pH values higher than 6.0 are mostly in the Y(OH)_3_, form, which is not soluble in aqueous solution, and therefore unable to form bonds with the polymer molecules. On this basis it was considered more reasonable to measure metal binding capacity of carrageenans regarding Y^3+^ and Pb^2+^ ions at pH values ranging from 2.0 to 6.0.

### 2.2. Effect of agitation period

**Figure 3:**
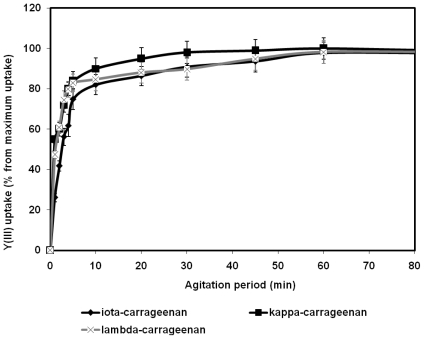
Effect of agitation period on the yttrium binding activity of carrageenans.

**Figure 4:**
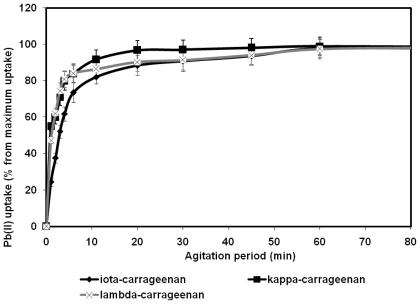
Effect of agitation period on the lead binding activity of carrageenans.

Duration of the agitation period is one of the most important factors influencing efficacy of any sorption system. It is known that the longer period of interaction between two components of the sorption system, the more quantity of the ions would be bound until the equilibrium concentration is achieved. In the present study the amount of the metal ions bound by a carrageenan sample was determined within strictly measured time intervals. Figures 3 and 4 display the effects of agitation period on the Y^3+^ and Pb^2+^ uptake by the three types of carrageenans. On the basis of these results the period required for equilibrium concentration between the polysaccharides and the metal ions to be achieved was found. In was determined that the amount of Y^3+^ ions as well as of Pb^2+^ ions bound by every type of carrageenan being studied dramatically increases within the first minutes of the agitation period and attains equilibrium in about 1 hour. During the first minute of the agitation period all carrageenans have bound 45%–53% of their maximum uptake under given conditions After 5 minutes of agitation the metal uptake of the carrageenans was close to the maximum possible saturation under the given conditions. At 60 minutes the plots shown in Figures 3 and 4 present a flat line. Therefore, based on the results of this part of experiment, the following studies were performed with the agitation periods varying from 60 to 120 minutes, which was considered sufficient for the equilibrium concentration to be achieved.

### 2.3. Effect of agitation rate

In other studies the rate of agitation has been found to have an influence on the chemical activity of the components of sorption system [9]. The following experiments were performed using the following stirring speeds: no stirring, 100, 200, 300, 400, and 500 rpm. The results obtained showed that the stirring rate does not influence the interaction processes between Y^3+^ and Pb^2+^ ions and all three types of carrageenans. Even if no stirring at all was applied in the batch sorption system, there was found no differences in the metal uptake values registered. Therefore, it may be concluded that water-soluble carrageenans possess sufficient affinity towards the metal ions and this interaction is not dependent on the velocity of the solution flow.

### 2.4. Equilibrium studies

The metal binding activity of ι-, λ-, and κ-carrageenans regarding Y^3+^ and Pb^2+^ ions as a function of equilibrium metal concentration was studied at pH 6.0 because previous experiments had showed that at such pH values all carrageenans exert their highest chemical activity. Interactions between the components of the sorption batch system generally result in the metal ions being removed from the solution and hence their concentration on the active binding sites of the polysaccharide increased, until the remaining ions in the solution are in the dynamic equilibrium with the ions bound to the sorption centers. Therefore, there is a strictly defined distribution of the bound and free metal ions in the sorption system, which can be expressed by one or more isotherms [9]. Figures 1 and 2 show the sorption curves indicating the amount of Y^3+^ and Pb^2+^ ions, respectively, bound to the carrageenan molecules increasing with rise of the equilibrium metal concentration in solution. For description of the interaction between sorbent and the metal ions being bound the sorption isotherm plotting is usually used. The isotherms are characterized by the initial region, which is represented as being concave to the concentration axis and then the isotherm reaches a plateau. In our study three sorption models were used as follows.

The Langmuir model is most often used to describe equilibrium sorption process characterizing by monolayer sorption with a finite number of identical sites. Presumably, binding of metal by the carrageenan molecule is associated with formation of identical active centers, each of which can interact with one metal ion. Such a mechanism corresponds to this model. The Langmuir equation is given by:

[1] $$\text{Q}={\text{q}}_{\max}{\text{bC}}_{\text{e}}/1+{\text{bC}}_{\text{e}}$$

where q_max_ is the maximum sorption at monolayer (mg·g^−1^), C_e_ is a final equilibrium concentration of metal ions in solution, *q* is the amount of metal ions bound per unit weight of a carrageenan at final equilibrium concentration (mg·g^−1^), *b* is the Langmuir constant related to the affinity of binding sites (mL·mg^−1^) and is considered as a measure of the energy of sorption. The following linearized plot of the Langmuir equation was used in this study:

[2] $${\text{C}}_{\text{e}}/{\text{q}}_{\text{e}}=({\text{C}}_{\text{e}}/{\text{q}}_{\max})+1/({\text{q}}_{\max}\text{b})$$

The Freundlich equation is commonly used for description of processes based on sorption through heterogeneous active centers. We can assume that the active sites of carrageenan interacting with metal ions may contain various numbers of hydroxyl groups. Thus, this model would better fit for describing such processes. The Freundlich equation is given by:

[3] $$l{\text{ogq}}_{\text{e}}={\text{logK}}_{\text{F}}+\frac{1}{\text{n}}{\text{logC}}_{\text{e}}$$

where *K_F_* and *n* are Freundlich constants indicating sorption capacity (mg·g^−1^) and intensity, respectively. *K_F_* and *n* can be determined from linear plot of log q_e_ against logC_e_.

The Brunauer-Emmett-Teller (BET) model is used to describe multilayer sorption. In this case the active centers of carrageenans probably may interact with more than one metal ion. In this case this model would suitably describe such processes. The BET equation is given by:

[4] $$\frac{C_{e}}{(C_{0}-C_{e})q_{e}}=\left(\frac{1}{{Bq}_{\max}}\right)+\left(\frac{B-1}{{Bq}_{\max}}\right)\left(\frac{C_{0}}{C_{e}}\right)$$

where q_max_ is the maximum uptake at monolayer (mg·g^−1^), *C_e_* is the equilibrium concentration of metal ions (mg·L^−1^), *C_0_* is the saturation concentration of the solute (mg·L^−1^), *q_e_* is the amount of metal ions bound per unit weight of a carrageenan at equilibrium concentration (mg·g^−1^) and *B* is the BET constant expressive of the energy of interaction with surface.

**Figure 5:**
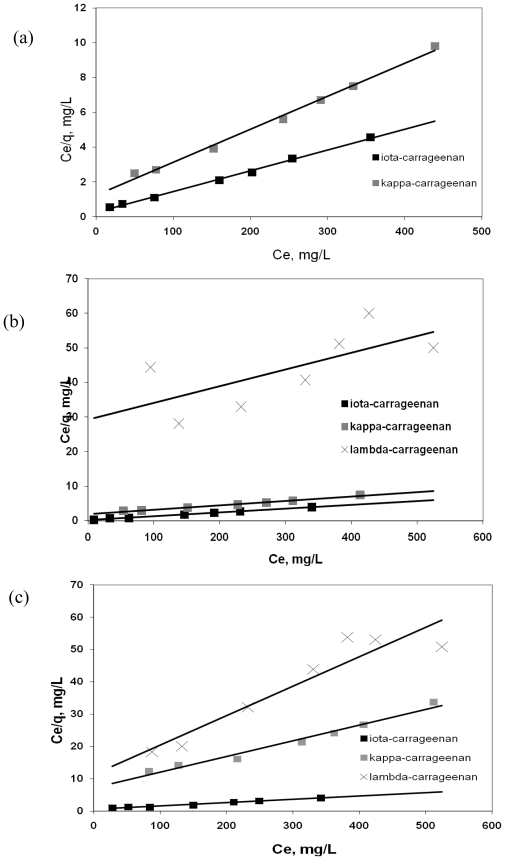
Langmuir plot for the sorption of Y^3+^ by carrageenans at various pH values of solution (a) pH = 2.0; (b) pH = 4.0; (c) pH = 6.0.

**Figure 6:**
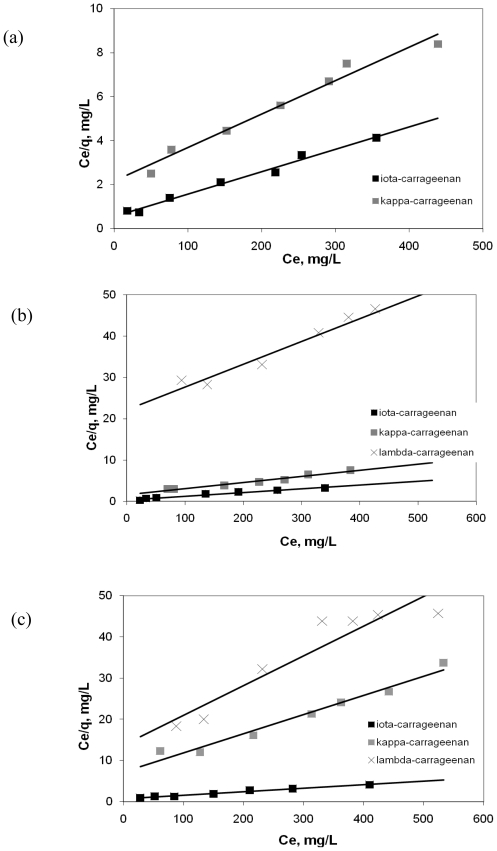
Langmuir plot for the sorption of Pb^2+^ by carrageenans at various pH values of solution (a) pH = 2.0; (b) pH = 4.0; (c) pH = 6.0

Calculated results of the Langmuir, Freundlich and BET isotherms are given in Tables 1 and 2. Table 1 shows the results of Y^3+^ binding by carrageenans; Table 2 depicts the results of interactions between Pb^2+^ and carrageenans. The results show that the binding of Y^3+^ and Pb^2+^ by ι- and κ-carrageenans were better correlated (*R^2^* *>* 0.96) with the Langmuir equation as compared to Freundlich and BET equations for the given range of concentration. This can be explained by the presence of a finite number of homogenous binding sites on the carrageenans presented as the free active hydroxyl groups, which is the basic condition of the Langmuir sorption model [9]. The results obtained in the experiments with λ-carrageenan were not considered significant after evaluation by application of all three models because the metal binding capacity of this type of carrageenan was too low and the values varied due to the errors of the methods applied. According to the calculated Langmuir parameters obtained from the plot of C/q *vs.* C (Figures 5 and 6), the highest binding capacity is typical of ι-carrageenan, which is characterized by the lowest degree of sulfation. This was proved by the highest value of the q_max_ as well as of the coefficient *b* that is related to the apparent energy of sorption. Coefficient *b* for ι-carrageenan was much greater than that of other types of carrageenans. Also it should be noted that both Langmuir parameters q_max_ and *b* are reversely altered according to the changes of the pH of the sorption system. q_max,_ reflecting the number of active centers was highest at pH 6.0 whereas *b* was highest at the acid values of pH. Presumably it may be explained by the fact that carrageenans are more stable at the neutral pH values and thus they can form strong bonds between their molecules and metal ions. Comparison of values of coefficient *b* at the same pH values showed the parameters of the metal binding activity of κ-carrageenan was much lower than those of ι-carrageenan. And also these parameters were pH dependent. λ-carrageenan characterized by the high content of sulfate groups [3] was found to exert the slightest metal binding activity regarding both metal ions studied. Binding capacity of this sample at the pH 4.0 and 6.0 was slightly higher than zero but at the pH 2.0 it even could not be determined. Therefore, ι-carrageenan was considered to have highest metal binding capacity.

The essential features of a Langmuir isotherm can be expressed in terms of a dimensionless constant separation factor, *R*_L_ that is used to predict if an adsorption system is “favorable” or “unfavorable”. The separation factor, *R*_L_ is defined by:

[5] $$R_{\text{L}}=1/(1+{\text{bC}}_{0})$$

where *C*_0_ is the initial metal concentration (mg·mL^−1^) and *b* is the Langmuir adsorption equilibrium constant (mL·mg^−1^).

The results of the *R_L_* factor calculation (Tables 3 and 4) showed that based on the effect of separation factor on isotherm shape, the *R_L_* values of all carrageenans studied, even λ-carrageenan, were in the range of 0 *< R_L_* *<* 1, which indicates that the binding of Y^3+^ and Pb^2+^ by these substances is favorable. R_L_ values for ι-carrageenan were between 0 and 1, but they were significantly different from those of other types of carrageenans, suggesting the lower binding capacity of these substances towards Y^3+^ and Pb^2+^. The obvious mechanism of sorption is related to the formation of covalent and hydrogen bonds between the metal ions and non-sulfated hydroxyl groups and hydrogen atoms located on the carrageenan molecules and acting as the binding centers. Results obtained show that intensity of binding processes and sorption capacity does not depend on solubility or other physicochemical parameters of the compound studied but closely relates to the number of the sulfated groups in its structure. The q_max_ parameter of the Langmuir model indicating the number of active binding sites of molecules shows that the lower the degree of sulfation of the carrageenan molecule is the more active sites on the hydroxyl groups are taking part in the process of the metal binding.

Changes of the *b* coefficient reflecting affinity of the carrageenans to metal ions were also strongly correlated to the amount of the sulfate groups; the highest values of affinity were typical of ι-carrageenan. A similar tendency was found after evaluation of the Freundlich equation parameters; even though they were considered less significant. The values of the *R^2^* results for the lead binding capacity of carrageenans varied from 0.92 to 0.95; therefore, these results can be discussed if related to the values of the Langmuir model. Combining these data with the value of the rate of sorption found in experiments the main mechanism is obviously chemisorption. In other words metal binding activity is caused by formation of the chemical covalent and ionic bonds between metals and the carrageenan molecules. These bonds are mush stronger than forces of the physical adsorption and provide creation of metal-polysaccharide complexes.

The results obtained through this study suggest that carrageenans, in particular, ι-carrageenan possess relatively high metal binding activity. Such a property may be useful for creation of cheap and effective medicines purposed for removal of various bivalent and trivalent ions from the human body. Such materials can also be successfully used for prevention of the entry of metal ions into the human body with inhaled air or consumed food and water. In other words, ι-carrageenan and probably κ-carrageenan can be used for elaboration of drugs purposed for treatment and prevention of the chronic metal poisoning in people exposed to metal ions in their local environments. Carrageenans may also be used as a base for creation of the targeted drug delivery systems for treatment of tumor-related diseases. One of the perspective directions in this field of medicine is development of the system providing targeted delivery of Y^90^ to malignant tumors [7]. From this point of view the non-starch polysaccharides, including carrageenans, may be considered as the perspective compounds for creation of the antitumor drug systems.

## 3. Conclusions

The chemical structure and physicochemical properties of the different carrageenans dramatically influence the metal binding activity of these compounds. The main parameter affecting metal uptake exerted by carrageenan is the sulfate group content. The lower the amount of sulfate groups is the more metal ions can be bound to the active sites of the carrageenan. Therefore, ι-carrageenan possesses highest metal binding activity

The pH change in the solution under some conditions affects the dynamics of the binding process. Binding of the bivalent ions by ι- and κ-carrageenans is lower under low pH values, whereas interaction with trivalent yttrium ions is not altered by the pH changes, except for the binding capacity of κ-carrageenan, which is characterized by a moderate amount of sulfate groups in its molecule. The optimum pH range for binding of the metal ions by κ-carrageenan is 4.0–6.0. The rate of the process providing the equilibrium concentration to be achieved is 60 minutes.

Equilibrium studies showed that carrageenans are favorable materials for binding and removal of metal ions from aqueous solution. On the base of results obtained the most preferable substance is ι-carrageenan with no sulfate groups in its molecule.

## 4. Experimental Section

### 4.1. Materials

ι-, λ-, and κ-Carrageenans were purchased from Sigma-Aldrich (USA). Carrageenan preparations contained individual types of carrageenans with no additives. Molecular weight of the carrageenan samples studied was approximately 200,000 Da. All other chemicals were of the highest quality available. Distilled water was used throughout.

### 4.2. Experimental procedures

0.1 M stock solutions of Y^3+^ and Pb^2+^ ions was prepared using analytical-reagent grade Y(NO_3_)_3_ and PbCl_2_, respectively. The stock solution was used for further preparation of the standard solutions with the metal concentrations required. The pH value of the standard solutions was controlled by addition of either 0.1 M HCl or 0.1 M NaOH and maintained at a constant pH. Batch sorption experiments were performed in 20 mL beakers and equilibrated using a magnetic stirrer. One mL aliquots of the standard solutions prepared by dilution of the stock solution were placed in 20 mL beakers containing 10 mL of 0.5% carrageenan solution. Then the total volume of the sorption system solution was made up to 20 mL by addition of distilled water. The system was equilibrated for a defined period of time then carrageenan polymers were separated from Y^3+^ and Pb^2+^ solution by using centrifugal force (3,000 g) for 10–20 minutes and subsequently filtered through a glass filter with a 100–120 μm pore size. In the experiments high-molecular carrageenan samples were used, thus they easily formed gel after interaction with the metal ions and were kept by the glass filter. Concentration of Y^3+^ and Pb^2+^ ions in the supernatant obtained was analyzed using an atomic absorption spectrophotometry method.

The effect pH of the media on interaction of carrageenans with Y^3+^ and Pb^2+^ ions was studied within a pH range from 2.0 to 6.0. The pH of the initial solution was adjusted to the required pH value using either 0.1 M HCl or 0.1 M NaOH. Carrageenans were equilibrated at the particular pH for about 60 min at 400 rpm and at an initial Y^3+^ and Pb^2+^ concentration of 0.6 g·L^−1^ using a bath controlled at 24 °C.

The effect of agitation period was also studied to determine the optimum duration of the chemical interaction between components of the system for sorption of Y^3+^ and Pb^2+^ ions. During this study 10 mL of 0.5% carrageenan solution were placed in 20 mL beakers and stirred by a magnetic stirrer at 400 rpm for 60 min with an initial Y^3+^ and Pb^2+^ concentration of 0.6 g·L^−1^ using a bath controlled at 24 °C. At the preset time intervals, the aqueous samples were taken out and the concentration of Y^3+^ or Pb^2+^ was assessed.

Sorption equilibrium studies were performed under the following conditions: duration of the agitation period 60 min, constant stirring 400 rpm, pH 6.0. It was concluded that execution of experiments at pH 6.0 is most reasonable because under such conditions all samples of the polysaccharides studied exert highest binding activity regarding metal ions and pH controlling procedure requires smallest amounts of HCl and NaOH. Controlled temperature of the batch sorption system was 24 °C. During the isotherm studies the amount of the carrageenan sample in the 20 mL beaker was constant (0.05 g) whereas initial concentration of Y^3+^ or Pb^2+^ varied within the range of 0.05–0.7 g·L^−1^. Each experiment was run at least in duplicate under identical conditions. A negative control experiment with no carrageenan added was simultaneously carried out to ensure that the metal removal was caused by the polysaccharide binding activity and not by the beaker or filter. The parameters obtained were subjected to a one-way analysis of variance using a software package SPSS (Statistical Package for Social Sciences) for Windows, version 11.0 with a confidence level of 95% (P < 0.05).

The metal accumulation (q) was determined as follows:

[6] $$q_{e}=\frac{(C_{0}-C_{e})\cdot V}{W}$$

where C_0_ is the initial metal concentration (mg·L^−1^), C_e_ is the final or equilibrium metal concentration (mg·L^−1^), V is the volume of the metal solution (mL), and W is the weight of the dry samples of carrageenan (g). The amount of the metal ions bound by the carrageenans was expressed in mg per g of the dry sorbent.

## Figures and Tables

**Table 1:** Langmuir, Freundlich and BET isotherm constants and correlation coefficients of Y^3+^ binding capacity of carrageenans.

Constants	ι-carrageenan			κ-carrageenan			λ-carrageenan		
	
	pH = 2	pH = 4	pH = 6	pH = 2	pH = 4	pH = 6	pH = 2	pH = 4	pH = 6
Langmuir model									
b, (mL mg^−1^)	0.045	0.057	0.017	0.015	0.006	0.006	-	0.001	0.008
q_max,_ (mg g^−1^)	84.0	90.1	99.0	52.6	83.3	20.8	-	20.4	10.9
R^2^	0.9970	0.9965	0.9818	0.9915	0.9848	0.9758	-	0.9893	0.9887

Freundlich model									
K_F,_ (mg g^−1^)	3.38	3.79	2.43	2.12	1.47	0.97	-	0.36	1.11
n	3.50	3.95	2.32	2.79	1.85	2.14	-	1.33	3.21
R^2^	0.8739	0.8976	0.8732	0.8939	0.9522	0.9173	-	0.8331	0.7967

BET model									
Q, (mg g^−1^)	33.3	41.6	24.0	9.8	55.5	1.21	-	0.18	0.3
B	1.11	1.00	0.93	2.00	0.10	4.33	-	0.18	0.26
R^2^	0.011	0.002	0.1648	0.1359	0.3678	0.0302	-	0.0421	0.6603

**Table 2:** Langmuir, Freundlich and BET isotherm constants and correlation coefficients of Pb^2+^ binding capacity of carrageenans.

Constants	ι-carrageenan			κ-carrageenan			λ-carrageenan		
	
	pH = 2	pH = 4	pH = 6	pH = 2	pH = 4	pH = 6	pH = 2	pH = 4	pH = 6
Langmuir model									
b, (mL mg^−1^)	0.047	0.042	0.032	0.017	0.014	0.012	-	0.007	0.012
q_max,_ (mg g^−1^)	132.2	154.6	178.0	67.3	103.0	36.9	-	26.1	13.9
R^2^	0.9834	0.9912	0.9900	0.9814	0.9741	0.9803	-	0.4890	0.8895

Freundlich model									
K_F,_ (mg g^−1^)	3.12	3.97	3.03	2.34	1.95	1.12	-	0.65	1.65
n	4.48	4.27	3.01	3.78	2.31	2.85	-	1.57	4.62
R^2^	0.9204	0.9412	0.9509	0.9300	0.9549	0.9458	-	0.9310	0.9478

BET model									
Q, (mg g^−1^)	45.9	38.7	32.6	12.5	43.8	12.9	-	0.43	0.65
B	1.76	1.65	1.38	3.82	1.76	5.34	-	0.54	0.64
R^2^	0.0961	0.1533	0.1294	0.5342	0.2323	0.1264	-	0.1876	0.1543

**Table 3:** *R*_L_ values based on the Langmuir equation for Y^3+^ binding at pH = 6.

Y^3+^ initial concentration, mg·L^−1^	R_L_ value		
	ι-carrageenan	κ-carrageenan	λ-carrageenan
20	0.749	0.882	0.861
40	0.599	0.788	0.756
60	0.499	0.713	0.673
80	0.427	0.650	0.608
100	0.374	0.598	0.553

**Table 4:** *R*_L_ values based on the Langmuir equation for Pb^2+^ binding at pH = 6.

Pb^2+^ initial concentration, mg·L^−1^	R_L_ value		
	ι-carrageenan	κ-carrageenan	λ-carrageenan
20	0.609	0.781	0.877
40	0.438	0.641	0.781
60	0.342	0.543	0.704
80	0.281	0.472	0.641
100	0.237	0.417	0.588
